# The challenges of the pandemic and the vaccination against covid-19 in pediatric patients with kidney disease

**DOI:** 10.1590/2175-8239-JBN-2022-0081en

**Published:** 2022-10-24

**Authors:** Emília Maria Dantas Soeiro, Maria Goretti Moreira Guimarães Penido, Lilian Monteiro Pereira Palma, Nilzete Liberato Bresolin, Eduardo Jorge da Fonseca Lima, Vera Hermina Kalika Koch, Marcelo de Sousa Tavares, Lucimary Sylvestre, Rejane de Paula Bernardes, Clotilde Druck Garcia, Maria Cristina de Andrade, Arnauld Kaufman, Charles Yea Zen Chow, Suelen Bianca Stopa Martins, Suzana Friedlander Del Nero Camargo

**Affiliations:** 1 Instituto de Medicina Integral Professor Fernando Figueira, Recife, PE, Brazil.; 2 Santa Casa de Belo Horizonte, Centro de Nefrologia, Unidade de Nefrologia Pediátrica, Belo Horizonte, MG, Brazil.; 3 Universidade Estadual de Campinas, Departamento de Pediatria, Campinas, SP, Brazil.; 4 Universidade Federal de Santa Catarina, Florianópolis, SC, Brazil.; 5 Hospital das Clínicas da Faculdade de Medicina da USP, Instituto da Criança e do Adolescente, São Paulo, SP, Brazil.; 6 Hospital Pequeno Príncipe, Curitiba, PR, Brazil.; 7 Clínica Nefrokids, Curitiba, PR, Brazil.; 8 Universidade Federal de Ciências da Saúde de Porto Alegre, Santa Casa de Porto Alegre, Serviço de Pediátrica, Porto Alegre, RS, Brazil.; 9 Universidade Federal de São Paulo, Escola Paulista de Medicina, São Paulo, SP, Brazil.; 10 Instituto de Puericultura e Pediatria Martagão Gesteira, Rio de Janeiro, RJ, Brazil.; 11 Universidade Federal do Rio de Janeiro, RJ, Brazil.; 12 Hospital Federal dos Servidores do Estado do Rio de Janeiro, RJ, Brazil.; 13 Grupo Assistência Médica Nefrológica, Rio de Janeiro, RJ, Brazil.; 14 Hospital do Rim, São Paulo, SP, Brazil.

**Keywords:** Vaccines, Covid-19, Hemolytic-Uremic Syndrome, Nephrotic Syndrome, Renal Insufficiency, Chronic, Kidney transplantation, Vacinas, Covid-19, Síndrome Hemolítico-Urêmica, Síndrome Nefrótica, Insuficiência Renal Crônica, Diálise, Transplante de Rim

## Abstract

The covid-19 vaccine confers direct protection and reduces transmission rates of the virus and new variants. Vaccines from Pfizer/BioNTech and CoronaVac have been cleared for children in Brazil. They are safe, effective, and immunogenic. There are no known complications associated with the use of steroids or vaccines in pediatric patients with covid-19 and nephrotic syndrome. With or without immunosuppression, these patients are not at increased risk of severe covid-19, and steroids are safe for them. A milder form of covid-19 occurs in patients with chronic kidney disease without the need for hospitalization. The vaccine response may be reduced and/or the duration of antibodies after vaccination may be shorter than in the general population. However, considering risk of exposure, vaccination against covid-19 is recommended. It is believed that patients with hemolytic-uremic syndrome are at higher risk of severe covid-19. Vaccination is recommended, although specific data on the safety and efficacy of the covid-19 vaccine are limited. There is agreement that the benefits of induced immunity outweigh the risks of immunization. Vaccination against covid-19 is recommended for children and adolescents needing kidney transplantation or who have undergone transplantation. These patients present decreased immune response after vaccination, but immunization is recommended because the benefits outweigh the risks of vaccination. Current recommendations in Brazil stipulate the use of the messenger RNA vaccine. This paper aims to provide pediatric nephrologists with the latest knowledge about vaccination against covid-19 for children with kidney disease.

## Resumo

A vacina covid-19 confere proteção direta, reduz as taxas de transmissão do vírus e de novas variantes. No Brasil, estão liberadas para a população pediátrica as vacinas Pfizer/BioNTech e a CoronaVac, ambas seguras, eficazes e imunogênicas. Pacientes pediátricos com síndrome nefrótica e covid-19 têm curso clínico regular sem complicações relacionadas ao uso de esteroides ou vacinas. Esses pacientes, com ou sem imunossupressão, não apresentam maior risco de covid-19 grave e o tratamento com esteroides é seguro. Os pacientes com doença renal crônica têm covid-19 mais leve, sem necessidade de hospitalização. A resposta vacinal pode ser reduzida e/ou a duração dos anticorpos pós-vacinação pode ser menor do que na população geral. Entretanto, a vacina covid-19 está recomendada, considerando o risco de exposição. Acredita-se que pacientes com síndrome hemolítico-urêmica teriam maior risco de covid-19 grave. A vacina é recomendada, embora dados específicos sobre segurança e eficácia da vacina covid-19 sejam limitados. Há concordância que os benefícios da imunidade induzida superam quaisquer riscos da imunização. A vacina covid-19 é recomendada para crianças e adolescentes candidatos ao transplante renal ou já transplantados. Esses pacientes têm resposta imunológica reduzida após a vacina, entretanto ela é recomendada porque os benefícios superam qualquer risco dessa vacinação. A recomendação atual no Brasil é a vacina de tecnologia RNA mensageiro. O objetivo deste documento é levar aos nefrologistas pediátricos os conhecimentos mais recentes sobre a vacinação contra contra-19 em crianças com doenças renais.

## INTRODUCTION

Covid-19 was first detected in December 2019 in Hubei (Wuhan) province, China. The virus has spread rapidly around the world, and by March 2022, 29 million cases of covid-19 and 652,000 deaths from the disease had been confirmed in Brazil^(1)^. In that same period, 6,531 cases of pediatric severe acute respiratory syndrome due to covid-19 and 1,503 cases of multisystem inflammatory syndrome in children with 93 deaths had been confirmed^(2,3)^.

A large proportion of children with covid-19 are asymptomatic or have mild symptoms, and the presence of comorbid conditions is considered a risk factor. A Brazilian study showed that 41% of children admitted to intensive care units had comorbid conditions^(4)^.

There are few reports on the risk of severe disease from covid-19 in immunocompromised pediatric patients. Population studies have shown that children and adolescents are exposed to the virus in a similar way to adults and are potential vectors in disease transmission^(5)^.

The covid-19 vaccine confers direct protection, reduces the rates of virus transmission and the emergence of new variants^(6)^. Lv et al. have demonstrated the safety, efficacy and immunogenicity of these vaccines in healthy pediatric populations. Adverse events are rare and mild, and benefits of vaccination outweigh the risks^(7)^.

The Pfizer/BioNTech vaccines (BNT162b2), authorized for children aged five years and older, and CoronaVac, authorized for children aged six years and older, are currently approved in Brazil. CoronaVac (Sinovac) is a vaccine with inactivated virus. The Pfizer-BioNTech covid-19 (BNT162b2) vaccine is a lipid nanoparticle of nucleoside-modified mRNA that enables the expression of SARS-CoV-2 protein S on the cell surface. It causes the activation of cytotoxic and helper T-cells and induction of humoral immunity, thereby producing neutralizing antibodies. Both vaccines are safe, effective, and immunogenic.

The most common adverse events in children and adolescents are injection site pain, fever, headache, and fatigue. Most of these events were not serious and deaths have not been reported^(7)^. Rare cases of myocarditis and/or pericarditis have been reported in association with the administration of the second dose of the covid-19 BNT162b2 mRNA vaccine after a short interval from the first dose (< 30 days), but no deaths have been attributed to these complications^(7)^.

**Chart 1:**
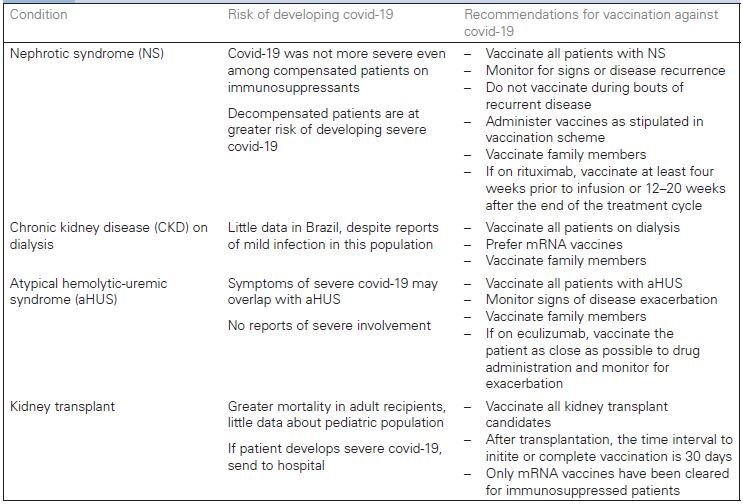
Risk of SARS-CoV-2 infection and recommendations for vaccination against covid-19 for each category of pediatric patient with kidney disease

Chart 1shows the risks of SARS-CoV-2 infection and the recommendations for the vaccination against covid-19 for each category of pediatric patients with kidney disease.

### Covid-19 and Covid-19 Vaccination in Children and Adolescents with Nephrotic Syndrome (NS)

Most children with idiopathic NS relapse or are steroid-dependent, and require chronic use of immunosuppressants. Urinary loss of endogenous antibodies during NS decompensation and immunosuppressant therapy increase the risk of infections^(8)^. Evidence points to immune system dysregulation involving B and T cells as part of the pathophysiology of NS, suggesting that vaccines may promote disease recurrence via the induction of immune response.

To date, there have been few reports of NS associated with covid-19 infection. A systematic review about covid-19 in patients with NS concluded that, with or without immunosuppressant therapy, patients were not at increased risk of severe covid-19, steroid treatment was safe, and the incidence of disease recurrence remained unchanged^(9)^. On the other hand, a study performed in New Delhi showed that patients with decompensated NS had a sixfold risk of developing severe complications during covid-19, such as severe acute kidney injury, shock, respiratory failure, encephalopathy, or death10. Cases of NS from minimal injuries triggered after vaccination against covid-19 involving adults and one adolescent have been reported^(8,11)^. Recommendations for vaccination are mostly based on expert opinions, considering the lack of controlled studies.

#### Immunosuppressant therapy for children and adolescents during the pandemic^9^

- Continue ongoing treatment, advising parents to report SARS-CoV-2 infection or related symptoms.
- Initiate or intensify immunosuppressant therapy as needed, without concerns related to covid-19.
- These patients do not require more stringent protective measures compared to their healthy peers.

#### Covid-19 infection in children and adolescents with NS

- For children with covid-19 in remission, treatment must be the same as the one given to healthy children and preventive hospitalization is not needed. Signs of recurrence must be monitored and, in cases of severe disease, hospitalization and reduction of immunosuppressant therapy must be considered.
- In cases of mild or asymptomatic infection, maintain ongoing treatment with immuno­suppressants; immediate hospitalization should be avoided. Monitor for signs of recurrence.

#### NS recurrence in children and adolescents

- Recurrent disease is treated with corticosteroids; there is no reason to delay the initiation of therapy.
- For covid-19-related recurrent disease, the usual protocol must be enforced.

#### Recommendations regarding covid-19 vaccines for children and adolescents with NS

- Vaccinate all patients with NS, following the age limits established by regulatory agencies.
- Signs of recurrence must be monitored after vaccination;
- Vaccines must not be administered to individuals with recurring disease.
- Every immunosuppressed patient over the age of 12 must have the third dose of the vaccine and receive the fourth dose four months later.
- In the case of ongoing anti-CD20 therapy (rituximab), vaccination must be postponed for at least six months after treatment cessation.

#### Covid-19 Vaccines for Children and Adolescents with NS on Rituximab 

The response to vaccination in patients taking rituximab is reduced. Thus, properly timing the administration of vaccines is necessary. Extending the interval between doses or discontinuing rituximab infusions allows immature B-cells to recover and proper vaccine response while levels of memory (pathogenic) B-cells remain low. An alternative strategy is to vaccinate patients at least four weeks prior to rituximab infusion or 12 to 20 weeks after the end of the infusion cycle. Monitoring the effect of rituximab from CD19 lymphocyte levels allows the discontinuation of the drug in asymptomatic and selected patients, which allows the definition of the time needed to improve response to vaccination. Rituximab infusions can be resumed four weeks after completing the vaccination scheme^(12,13)^.

### Covid-19 and Vaccination Against Covid-19 in Children and Adolescents with Chronic Kidney Disease on Dialysis

There are few studies about covid-19 in pediatric patients with chronic kidney disease (CKD) and on dialysis (peritoneal dialysis, PD, or hemodialysis, HD). These studies report the occurrence of milder disease and no need for hospitalization^(13,14)^. On the other hand, Aimen et al. found that CKD was the most common comorbid condition in symptomatic children and adolescents. One of the three deaths reported in their study involved a patient on PD^(15)^. In Brazil, one of the countries with the highest number of deaths by covid-19 in the pediatric age group, there is no specific data about patients on dialysis.

The usual vaccination schedule is recommended for children and adolescents, with special attention to vaccines with attenuated virus, which are contraindicated after renal transplantation. Vaccine response in CKD patients may be reduced and/or antibodies may be active for shorter periods of time than in the general population^(16)^. Nonetheless, given the risk of exposure, vaccination against covid-19 is recommended. It is also important that family members of dialysis patients get the full vaccination regimen, especially those with children under five years of age.

There is no evidence regarding the efficacy of covid-19 vaccines in pediatric patients on dialysis. In the Netherlands, the RECOVAC consortium (REnal patients COvid-19 VACcination), a prospective cohort study including dialysis patients older than 18 years, was organized to evaluate the efficacy of covid-19 vaccines in patients with CKD stages 4 and 5 and after kidney transplantation, comparing them with unvaccinated controls^(17)^.

Zitt et al. evaluated the safety and immunogenicity of the BNT162b2 vaccine in HD patients. They found local reactions in 38% after the first dose, while 29.2% had mild reactions after the second dose (2.1% moderate; 2.1% serious adverse events). Systemic events occurred rarely, and the most frequent were diarrhea (4% mild; 4% moderate) and fatigue (8% mild). After the first dose, 42% developed adequate vaccine response as assessed by IgG levels against anti-SARS-CoV-2 spike protein^(18)^. After the second dose, seroconversion was observed in 97.2% and was correlated with prior hepatitis B seroconversion and age (younger patients). Patients who had local reactions tended to have higher levels of protective antibodies. Conversely, patients on immunosuppressants during the study had lower levels of protective antibodies^(18)^.

Shashar et al. discussed the administration of the third dose in individuals on HD. The authors observed that the group that received the booster, compared to controls, had higher levels of protective antibodies, despite being older and having a greater incidence of hypertension. Serologic response was inversely associated with levels of inflammation markers and malnutrition. A drop in protective antibodies levels was observed eight months after vaccination in the group that did not receive booster shots^(19)^. This observation contributed to the discussion of the need for a third dose in individuals on HD^(20)^. Angel-Korman et al. confirmed this need, while others have wondered whether vaccination of individuals on HD should be considered on an individual basis^(21,22)^.

Based on study findings, some medical societies have presented specific recommendations for pediatric patients on dialysis. The British Association for Pediatric Nephrology (BAPN) recommends that covid-19 booster be given only to adolescents with CKD older than 12 years^(23)^. The EUDIAL working group of the European Dialysis and Transplant Association (2021) stated that adult patients and children alike should be vaccinated against covid-19^(24)^.

#### Covid-19 infection in children and adolescents with CKD and on dialysis

- Use the same treatment given to healthy children without the need for preventive hospitalization. In case of severe symptoms, consider hospitalization.
- In case of mild or asymptomatic infection, maintain treatment; immediate hospitalization should be avoided.

#### Recommendations to vaccinate children and adolescents with CKD and on dialysis against covid-19

- Vaccinate all pediatric patients with CKD and on dialysis, following the age limits set by regulatory agencies.
- Vaccinate preferably with an mRNA vaccine, in accordance with age restrictions.
- Family members of patients on dialysis and with CKD must comply with the complete vaccination scheme, especially those with children under the age of five years.
- All immunosuppressed patients over 12 years of age must take the third dose of the vaccine and the fourth dose four months later.

### Covid-19 and Covid-19 Vaccination in Children and Adolescents with Atypical Hemolytic-Uremic Syndrome (aHUS)

aHUS is a microangiopathic disorder whose pathophysiology overlaps with the cytokine storm observed in severe covid-19^(25)^. This shared pathophysiology suggests that patients with aHUS are at increased risk of developing severe covid-19, regardless of the status of the treatment for aHUS, including individuals previously diagnosed with covid-19^(26)^. The recommendation is that these patients are immunized against covid-19^(27,28)^. Although specific safety and efficacy data on the Pfizer-BioNTech vaccine is limited, there is agreement that the benefits of induced immunity outweigh the risks tied to immunization^(29)^. In Brazil, the use of live inactive virus vaccines (CoronaVac/Sinovac) has not been authorized for immunosuppressed patients^(27)^. Since aHUS is a serious condition, it has been excluded from the Pfizer-BioNTech, Moderna, and AstraZeneca vaccine trials. Thus, it is unclear whether currently available vaccines are as effective for these patients as they were for the studied populations^(30,31)^. There is no data to suggest that the available vaccines are less effective or less safe for individuals with aHUS than for the general population.

#### Covid-19 infection in children and adolescents with aHUS

- Maintain treatment; no need for preventive hospitalization. In case of severe infection, consider hospitalization and discontinuation of treatment.
- In cases of mild or asymptomatic infection, maintain treatment; immediate hospitalization should be avoided.

#### Recommendations for covid-19 vaccination in children and adolescents with aHUS

- Vaccinate all pediatric patients with aHUS, following the age limits set by regulatory agencies.
- Children and adolescents with a history of severe allergic reaction to a previous dose of vaccine or to one of its components should not be vaccinated^(31)^.
- Family members of patients diagnosed with aHUS must comply with the full vaccination scheme, especially family members of children aged less than five years.
- In case of comorbid conditions, vaccination must be postponed in individuals with severe acute fever or acute infection.
- In case of mild infection and/or low fever, do not postpone vaccination.
- In patients with thrombocytopenia and coagulation disorders, the vaccine must be administered with caution as in other intramuscular injections, with risk of local hematoma.

Patients on eculizumab should be vaccinated as close as possible to the day of drug infusion (days before or days after) because of the theoretical possibility that such approach might reduce the chance of disease exacerbation related to vaccine administration^(29)^.

Covid-19 vaccines can be given concurrently or at any time before or after any other indicated vaccine^(29)^. This is a change from the previous recommendation, which called for a 14-day interval before or after receiving a covid-19 vaccine. The basis for this change in recommendation stems from general administrative guidance for vaccines and guidance from the US Advisory Committee on Immunization Practices (ACIP)^(28)^.

### Covid-19 and Covid-19 Vaccination in Children and Adolescents Undvergoing Kidney Transplantation

The covid-19 pandemic has negatively impacted pediatric transplantation in Brazil and affected areas such as outpatient care, monitoring, transdisciplinary care, medication, patient/family education/support, schooling, employment, and care of pediatric kidney transplant patients diagnosed with covid-19.

Vaccination against covid-19 is recommended for all individuals, including children and adolescents waiting for kidney transplantation or transplant patients, as authorized by the FDA (Food and Drug Administration) and recommended by the Brazilian Ministry of Health^(32)^. In suspected or confirmed cases, vaccination must not be performed during the quarantine period^(27,33)^. In Brazil, the current recommendation is to use the Comirnaty (Pfizer-BioNTech) messenger RNA (mRNA) vaccine for immunosuppressed patients with an ideal interval of eight weeks between the first and second doses, in individuals aged 5 to 17 years^(27)^.

The covid-19 vaccine causes reduced immune response in solid organ transplant recipients when compared to immunocompetent individuals^(34)^. Studies in adult recipients have shown that vaccination led to a reduction of almost 80% in the incidence of symptomatic covid-19 compared to unvaccinated recipients^(35)^. Unfortunately, studies in pediatric solid organ transplant recipients are limited. Qin et al. showed that 73% of pediatric patients had a positive antibody response after two doses of mRNA vaccine^(36)^. Experience with other vaccines has shown that they continue to provide substantial protection against infections and more severe disease in this vulnerable population and should be recommended before and after transplantation^(35)^. Considering this experience, covid-19 vaccination for all solid organ recipients is recommended^(37,38)^.

#### Covid-19 infection in pediatric kidney transplant recipients

- Maintain the same treatment given to healthy children, with no need for preventive hospitalization. In case of severe covid-19 infection, consider hospitalization.
- In case of mild or asymptomatic infection, maintain treatment; hospitalization should be avoided.

#### Recommendations vaccinating children and adolescents undergoing kidney transplantation against covid-19

- Vaccinate all pediatric kidney transplant patients, following the age limits set by regulatory agencies.
- Use preferably an mRNA vaccine, following age restrictions.
- Family members of renal transplant patients must comply with the full vaccination scheme, especially family members of children aged less than five years^(39)^.
- In suspected or confirmed cases, do not vaccinate during the quarantine period;
- Kidney transplant recipients should receive any of the available covid-19 vaccines based on age and eligibility criteria.
- The optimal time to begin vaccination or complete the vaccination scheme after transplantation is unclear. Experts recommend waiting at least one month after transplantation to allow for a more robust immune response.
- All immunosuppressed patients over the age of 12 must have the third dose of the vaccine and the fourth dose four months later.
- Contraindications to the mRNA vaccine for solid organ recipients are the same as the general population:
  - Hypersensitivity to the active ingredient or to any of the excipients of the vaccine.
  - Confirmed anaphylactic reaction to a previous dose of a covid-19 vaccine.
- Postpone vaccination in individuals with severe acute fever or acute infection. Mild infection and/or low-grade fever should NOT cause postponement of vaccination.
- In patients with thrombocytopenia and coagulation disorders, administer the vaccine with caution, as with other intramuscular injections, with risk of local hematoma;
- Do not postpone kidney transplantation for kidney transplant candidates. They can receive the vaccine and do not need to wait for the procedure.
- After transplant, the interval to start or complete the vaccination scheme is 30 days.
- Do not change the immunosuppressants in use; postpone vaccination if the patient has acute fever.
- After vaccination, wear face masks, practice social distancing, and clean hands frequently.

## FINAL CONSIDERATIONS

Covid-19 causes mild symptoms or is asymptomatic in the majority of pediatric patients with CKD, on immunosuppressants due to glomerular disease, or undergoing renal transplantation.

In this group of individuals, vaccination against covid-19 is very important, since it confers direct protection and prevention against the disease. Vaccines reduce virus transmission rates and the emergence of new variants. Adverse events are rare and mild, and the benefits outweigh the risks.

Immunocompromised patients may not develop sufficient immune response after two doses of the vaccine. Studies recommend additional vaccines to improve response. Acceptance of the covid-19 vaccine is necessary to limit the risk that the disease poses to patients^(40)^.
